# Multimorbidity and Depressive Symptoms and their Association with Self-Reported Health and Life Satisfaction Among Adults Aged ≥ 50 Years in Mexico

**DOI:** 10.1007/s10823-025-09521-4

**Published:** 2025-01-23

**Authors:** Alvaro García Pérez, Teresa Villanueva Gutiérrez

**Affiliations:** 1 Laboratorio de Investigación en Salud Pública, Facultad de Estudios Superiores (FES) Iztacala, Universidad Nacional Autónoma de México (UNAM), Tlalnepantla , Mexico; 2 Departamento de Atención a la Salud, Universidad Autónoma Metropolitana Xochimilco, CDMX, Mexico; 3 Facultad de Estudios Superiores (FES) Iztacala, Universidad Nacional Autónoma de México (UNAM), Avenida de los Barrios Número 1 Colonia Los Reyes Ixtacala Tlalnepantla, Estado de México, Tlalnepantla, C.P. 54090 Mexico

**Keywords:** Multimorbidity, Life satisfaction, Older adults, Self-reported health, Depressive symptoms

## Abstract

Identify the association between multimorbidity and depressive symptoms (DS) with self-reported health (SRH) and life satisfaction in a national sample of Mexican ≥ 50 years older adults. Data are drawn from the Mexican Health and Aging Study (MHAS), a cross-sectional study conducted in 2018 involving 14,230 older adults aged 50 years and older living in urban and rural areas of Mexico. Depressive symptoms were measured using the Center for Epidemiological Studies depression scale (CES-D) and life satisfaction using the Life Satisfaction Scale (LSS), examined both as a categorical and continuous variable. Logistic and Poisson regression analyses were used to assess the association adjusting for confounders. The prevalence of multimorbidity was 25.8%. Age (≥ 65 years) was significantly associated with increased odds of fair/poor SRH [OR = 1.17 (95% CI 1.09–1.27)]. Older adults with multimorbidity and high DS were more likely to present fair/poor SRH [OR = 7.83 (95% CI 6.48–9.45)]. Older adults with multimorbidity and high DS were 37% [RR = 1.37 (95% CI 1.35–1.40)] more likely to present low life satisfaction than those that did not present multimorbidity. Multimorbidity and high DS were found to be associated with fair/poor SRH and low life satisfaction. The early identification of factors related to multimorbidity, DS, and low life satisfaction are important in order to reduce possible complications and improve quality of life.

## Introduction

According to the World Health Organization (WHO), aging is defined as the result of the accumulation of a great variety of cellular and molecular damage over time, leading to reduced physical and mental abilities and a higher probability of ill health causing death (WHO, 2022). Global statistics show an increase in the number of people aged 60 years and over, which is projected to rise from the one billion recorded in 2020 to 1.4 billion by 2030 and 2.1 billion by 2050 (WHO, 2022), and life expectancy increasing from 66.8 years in 2000 to 73.4 years in 2019 (WHO, 2019).

In Latin America and the Caribbean, the proportion of the population aged 60 and over will have increased by 25 to 30% by 2050 (da Silva et al., 2021), while, in Mexico, this will nearly triple, increasing from 6.3% in 2010 to almost 23% by 2050 (Angel et al., 2017). With the incidence of many health problems increasing with age, evidence shows that older adults may experience various health conditions at the same time, while it has also been observed that almost all chronic conditions are related to aging (Mino-Leon et al., 2017).

Multimorbidity refers to the coexistence of two or more chronic conditions, which are frequently found in older adults (Salive, 2013; Wang et al., 2020; Ando et al., 2022) and are related to increased mortality, reduced functional state, increased use of medical services, increased consumption of medication, and reduced quality of health. Multimorbidity not only has negative consequences in terms of mortality, but also impacts physical and mental wellbeing, life satisfaction, quality of life, and SRH. It has been observed that presence of multimorbidity is related to a negative self-rated health; similarly, in older adults, it has been found that those who have a negative self-rated of their health have reported a higher percent of DS. (Bustos-Vázquez et al., 2017). Little is known about the extent to which multimorbidity might independently affects health and life satisfaction in the older persons.

SRH is a qualitative indicator of health that is easy to use via a subjective question: How would you describe your general health? The inclusion of this question can be of great significance, as it can be used to elicit indicators of a person’s real state of health. SRH is a reliable measure in health, various studies have demonstrated that lower self-reported health is associated with higher levels of both morbidity and mortality – especially among older adults (Whitmore et al., 2022). Other investigations have found factors other than multimorbidity that are associated with self-reported health including demographic (e.g., sex), health-related (e.g., depressive symptoms) (Camargo-Casas et al., 2018), and behavioral factors (Whitmore et al., 2022). However, little is known about how these factors related to self-reported health and life satisfaction or whether the relationship between multimorbidity changes in the presence of SRH and life satisfaction in older adults.

Life satisfaction is an indicator of quality of life that includes physical, mental, and social wellbeing. As physical and psychological problems increase with advancing age, it is difficult to achieve high levels of life satisfaction as one gets older (Papi & Cheraghi, 2021). Previous studies have identified links between multimorbidity and life satisfaction, indicating that adults with multimorbidity present lower levels of life satisfaction (Wister et al., 2016), while also finding that a lower level of life satisfaction is a risk factor for both chronic conditions and death (Rosella et al., 2019). Data obtained by the European Social Survey 2014 reveal a negative association between multimorbidity and both SRH and life satisfaction (Marques et al., 2018). Medical practice aims to preserve the quality of life through the prevention and treatment of diseases. In this sense, people with chronic diseases require evaluations regarding the improvement or deterioration of their functional status. Although several studies worldwide have focused on life satisfaction of older people, in Mexico there is little research on effects of multimorbidity and DS about levels of life satisfaction among older adults.

Multimorbidity and DS are problems that affect the Mexican population health and have been linked to aging process. In Mexico, an increase in the prevalence of chronic diseases such as diabetes, cardiovascular diseases, high blood pressure and cancer has been observed. People with multimorbidity have a higher risk of disability, hospitalizations, mental disorders such as depression, decreased quality of life, and death (Novak & Lozano Keymolen, 2023). Studying the relationship between both conditions and life satisfaction will allow us to identify priorities in health areas, as well as prevent and improve the health of those who suffer from them (Celik et al., 2018). Analyzing these relationships using recent cross-sectional data may help to obtain a better understanding of the impact of the multimorbidity and DS related to other factors found in older adults in Mexico. The aims of the analysis were: (I) Determine the prevalence of multimorbidity; and, (II) Identify the association between multimorbidity and depressive symptoms (DS) with self-reported health (SRH) and life satisfaction in a national sample of Mexican ≥ 50 years older adults. The hypothesis proposed by the present study is that given the complexity and heterogeneity of health state of older adults and chronic effects of aging, it could be thought that multimorbidity and DS are related to low life satisfaction and poor SRH in older adults.

## Materials and Methods

Type of research: The study had a cross-sectional design.

The present study used the MHAS-2018 wave as a nationally-representative cross-section of the Mexican population aged 50 and over from both urban and rural areas. This study has a national probabilistic sample representative of individuals 50 years or older; it was designed to understand the different determinants of aging. It began in 2001, with subsequent waves in 2003, 2012, 2015, 2018 and 2021. For this study, the fifth wave 2018 is being analyzed. The MHAS survey collects information on socio-demographic characteristics, health, self-reported health in various dimensions (chronic diseases, physical function, perceived global health, depression, and cognition); life satisfaction, and other topics for persons aged 50 and over in Mexico. MHAS was originally designed to prospectively analyze the impact of disease on health, function, and mortality for older adults living in Mexico.

The MHAS surveys are conducted under the supervision of coordinators from both Mexico and the United States and are partially funded by the National Institutes of Health and Mexico’s National Institute of Statistics and Geography ((INEGI). The MHAS databases are available at http://www.ENASEM.org. The research carried out on the MHAS databases by the present study ​adhered to the relevant ethical conditions for research on human beings and were approved by the corresponding ethics committee (University of Texas Medical Branch and, in Mexico, by INEGI and the National Institute of Public Health) (NIH R01AG018016). All participants signed an informed consent form. For this study, we considered the initial sample of 17,114 (100%) individuals from the fifth wave (2018); of these we analyzed 14,230 (83.14%), who were aged 50 years and more and answered the life satisfaction and SRH questions. 16.8% of participants were excluded due to missing information in the database.

### Dependent Variables

Both SRH and life satisfaction were selected as the main outcomes. SRH was evaluated by means of a standardized question asking *“Would you say your health is…?”*, with the responses ranked on a Likert scale of excellent/very good/good/fair/poor and categorized into (excellent/very good/good and fair/poor).

The present study used the Life Satisfaction Scale (LSS), a questionnaire included in the Spanish version of the MHAS 2018 and measuring subjective criteria for life satisfaction that correspond to five items evaluated via multiple-choice options. The original LSS scale includes seven multiple-choice answers ranging from 1 = Strongly disagree to 7 = Strongly agree. The MHAS academic committee discussed the need to adapt the response scale of the original LSS for use in the Mexican population and decided to reduce the response options to three choices: 1 = Agree; 2 = Neither agree nor disagree; and, 3 = Disagree. The scores ranged from 1 to 3, while the possible scores for the adapted LSS scale included in the MHAS ranged from 5 (high satisfaction) to 15 (low satisfaction). Moreover, analysis conducted on the reliability of the scale found good internal consistency and construct validity (α = 0.74) (López-Ortega et al., 2016). High values of the LSS reflect low life satisfaction.

### Independent Variables

Self-reported multimorbidity (number of chronic diseases out of diabetes, hypertension, heart attack, asthma, arthritis, and cancer), with a disease or condition considered present if the participant answered *“yes”* to the question *“Has a doctor or medical personnel ever diagnosed you with…?”* and the answers then grouped into (non-multimorbidity and multimorbidity); and, DS, which were evaluated using a nine-item scale modified from the Center for Epidemiological Studies’ depression scale (CES-D), with older adults reporting a score from 0 to 4 categorized as having low DS, while a score ≥ 5 indicated high DS (Aguilar-Navarro et al., 2007). The association between multimorbidity and DS was ascertained by grouping the two variables together into four categories: 0 = Without DS/without multimorbidity (reference group); 1 = Without DS/with multimorbidity; 2 = With DS/without multimorbidity; and, 3 = With DS/with multimorbidity.

### Covariates

Age (in years), categorized into (50–65 years and ≥ 65 years); sex (male/female); place of residence (urban/rural); years of education dichotomized into ≤ 9 years and > 9 years; smoking status, categorized into (never/current/former smoker); alcohol consumption, categorized into (yes/no/has never drunk alcohol); pain level, categorized into (no pain/mild/moderate/severe). Finally, the respondents were asked: *“in the last 12 months*, *how much effect do you think stress has had on your health?”*, It will be used in manuscript with following phrase: “health-related stress” with responses categorized into (very much/something and almost nothing/nothing).

### Statistical Analysis

Descriptive statistics were calculated for the entire sample (means, standard deviation and percentages) for Table 1. The relationship between independent variables and covariates, according to SRH, was tested by Pearson’s chi-squared test (Table 2). For the relationship between the independent variables, covariates and the dependent variable life satisfaction scale, the Mann-Whitney and Kruskal-Wallis tests were used. Because the life satisfaction variable does not meet the assumption of normality (Shapiro-Wilk W test z = 17.53, *p* < 0.001) (Table 3). The logistic regression model (Table 4—Model 1) was applied to ascertain the association between the dependent variable, SRH (0 = Excellent/Very good/Good or 1 = Fair/poor), and the independent variables adjusted for confounders, while the Odds Ratio (OR) was calculated to a 95% confidence interval (95% CI). Model diagnostic tests were conducted using the Hosmer-Lemeshow goodness-of-fit test. Poisson regression models (Table 4—Model 2) were also estimated with robust variance to determine whether low life satisfaction was associated with the independent variables, which were compared in terms of the rate ratios (RRs) and respective 95% CIs. Theoretically plausible interactions were also explored (particularly, between health-related stress and DS, and the variables health-related stress and multimorbidity). All analyses were conducted using Stata 15, with the results considered statistically significant at a two-sided *p* < 0.05.

**Table 1:** Characteristics for total sample of the older adults of Mexican Health and Aging Study (MHAS-2018)

Variables	Total (*n* = 14,230) % or Mean (SD)
**Age**	64.7 (± 10.4)
**Sex**	
Male	42.8
Female	57.2
**Age groups**	
50–64 years	52.0
≥65 years	48.0
**Place of Residence**	
Urban	80.1
Rural	19.9
**Years of education**	
≤ 9 years	80.8
> 9 years	19.2
**Smoking Status**	
Never	67.6
Current	11.5
Former Smoker	20.9
**Alcohol Consumption**	
No	61.3
Yes	27.7
Never drank alcohol	11.0
**Depressive Symptoms**	
Low	70.2
High	29.8
**Pain level**	
No pain	60.1
Mild	13.4
Moderate	17.2
Severe	9.3
**Multimorbidity**	
No	74.2
Yes	25.8
**Diabetes**	
No	74.8
Yes	25.2
**Hypertension**	
No	55.1
Yes	44.9
**Arthritis**	
No	87.5
Yes	12.5
**LSS score**	7.42 (± 3.0)
**SRH**	
Excellent/very good	7.5
Good	28.9
Fair	53.9
Poor	9.7

**Table 2:** Characteristics of the older adults by Self-Reported Health of Mexican Health and Aging Study (MHAS-2018) (***n*** **=14,230**)

	Self-Reported Health (SRH)		
	Excellent/Very good/Good (*n* =**5,173**)	Fair/poor (*n* =**9,057**)	**p**
	**%**	**%**	
**Age**			
50–64 years	58.9	48.1	< 0.001
≥ 65 years	41.1	51.9	
**Sex**			
Male	48.5	39.6	< 0.001
Female	51.5	60.4	
**Place of Residence**			
Urban	84.6	77.6	< 0.001
Rural	15.4	22.4	
**Years of education**			
≤ 9 years	66.7	88.9	< 0.001
> 9 years	33.3	11.1	
**Smoking Status**			
Never	64.1	69.6	< 0.001
Current	13.2	10.6	
Former Smoker	22.7	19.8	
**Alcohol Consumption**			
No	57.3	63.7	< 0.001
Yes	32.6	24.8	
Never drank alcohol	10.1	11.5	
**Pain level**			
No pain	77.9	50.0	< 0.001
Mild	9.6	15.5	
Moderate	9.6	21.5	
Severe	2.9	13.0	
**Multimorbidity**			
No-multimorbidity	76.0	43.8	< 0.001
Only multimorbidity	11.2	22.9	
Only Depressive Symptoms (DS)	10.2	17.2	
Multimorbidity and DS	2.6	16.0	
**Health-related stress?**			
Very much/Something	95.1	85.7	< 0.001
Almost nothing/Nothing	4.9	14.3	

**Table 3:** Mean comparisons of the life satisfaction scale scores in the older adults of Mexican Health and Aging Study (MHAS-2018) (*n* = 14,230)

	Mean (SD)	*p*	Median	*p*
**Age** ^*^				
50–64 years	7.15 (2.8)	< 0.001	6	< 0.001
≥ 65 years	7.70 (3.1)		7	
**Sex** ^*^				
Male	7.4 (2.8)	0.599	6	0.008
Female	7.4 (3.1)		6	
**Place of Residence** ^*^				
Urban	7.3 (3.0)	< 0.001	6	< 0.001
Rural	7.7 (2.9)		7	
**Years of education** ^*^				
≤ 9 years	7.6 (3.0)	< 0.001	7	< 0.001
> 9 years	6.6 (2.7)		5	
**Smoking Status** ^**^				
Never Smoker	7.4 (3.0)	0.158	6	0.215
Current Smoker	7.2 (2.8)		6	
Former Smoker	7.4 (3.0)		6	
**Alcohol Consumption** ^**^				
No	7.5 (3.0)	0.001	6	< 0.001
Yes	7.2 (2.8)		6	
Never drank alcohol	7.7 (3.1)		7	
**Pain level** ^**^				
No pain	7.3 (2.8)	0.001	6	0.067
Mild	7.5 (3.0)		6	
Moderate	7.6 (3.2)		6	
Severe	7.8 (3.4)		7	
**Multimorbidity** ^**^				
No-multimorbidity	6.8 (2.2)	0.001	6	< 0.001
Only multimorbidity	6.9 (2.2)		6	
Only Depressive Symptoms (DS)	9.0 (4.1)		7	
Multimorbidity and DS	9.4 (4.1)		8	
**Health-related stress?** ^*^				
Very much/Something	7.4 (2.9)	0.168	6	0.687
Almost nothing/Nothing	7.7 (3.4)		6	

^*^Mann–Whitney Test, ^**^Kruskal–Wallis test, SD: Standard Deviation

**Table 4:** Logistic regression model for self-reported Health (SRH) **(model 1)**, and Poisson regression analysis for life satisfaction scale **(model 2)** in older adults of Mexican Health and Aging Study (MHAS-2018) (*n* =**14,230**)

	Model 1		Model 2	
	Fair/poor SRH		Low Life Satisfaction	
Variables	Adjust OR (95%CI)^*^	*p*	Adjust RR (95%CI)^**^	*p*
**Sex (Male ref.)**				
Female	1.05 (0.97–1.13)	0.206	0.96 (0.95–0.97)	0.001
**Age (50–64 years ref.)**				
≥ 65 years	1.17 (1.09–1.27)	< 0.001	1.02 (1.00–1.03)	0.003
**Place of Residence (Urban ref.)**				
Rural	1.36 (1.24–1.50)	< 0.001	1.05 (1.03–1.06)	< 0.001
**Years of Education (> 9 years ref.)**				
≤ 9 years	3.33 (3.02–3.67)	< 0.001	1.09 (1.07–1.11)	< 0.001
**Multimorbidity/DS (No multimorbidity not low DS ref.)**				
Only multimorbidity	2.81 (2.52–3.12)	< 0.001	1.02 (1.00–1.03)	0.018
Only DS	2.72 (2.43–3.05)	< 0.001	1.31 (1.29–1.34)	< 0.001
Multimorbidity/high DS	7.83 (6.48–9.45)	< 0.001	1.37 (1.35–1.40)	< 0.001
**Health-related stress? (Nothing ref.)**				
Very much/Something	2.24 (1.91–2.63)	< 0.001	0.98 (0.96–1.00)	0.100

-DS = Depressive Symptoms

-Logistic Regression Model: ^*^OR = Odds Ratio; CI = Confidence Interval. Log likelihood = -8058.7433

Hosmer-Lemeshow = 0.5535

-Poisson regression analysis ^**^RR = Rate Ratio, Log likelihood = -33697.932

## Results

Table 1 presents the characteristics of the study sample. The most prevalent chronic diseases were diabetes (25.2%), hypertension (44.9%), and arthritis (12.5%). The prevalence of multimorbidity (≥ 2 chronic diseases) was 25.8%, corresponding to 20.0% and 30.1% (*p* < 0.001) of male and female participants, respectively. Furthermore, as age increased, the prevalence of multimorbidity increased, breaking down as 17.4% (50–59 years), 28.0% (60–69 years), and 33.3% (≥ 70 years). The mean LSS score was 7.42 (± 3.0), with 61.9% presenting a score of ≥ 6 points.

Table 2 presents the results of the bivariate analysis conducted between the independent variables and the SRH, with 60.4% of female and 39.6% of male respondents rating their health as fair/poor (*p* < 0.001). More older adults with ≤ 9 years of education rated their health as fair/poor than those with > 9 years of education (88.9% vs. 11.1%; *p* < 0.001). Moreover, 22.9% of older adults with multimorbidity and 16.0% with multimorbidity/high DS rated their health as fair/poor.

Table 3 shows the comparison between the LSS scores and independent variables, with statistically significant differences observed. Older adults with multimorbidity/high DS presented a low life satisfaction score than adults without multimorbidity (9.4 vs. 6.8; *p* < 0.001).

### Multivariable Analysis

Table 4—Model 1 presents the results of the multivariate logistic regression analysis conducted, showing that age, place of residence (rural/urban), years of education (≤ 9 years), multimorbidity/high DS, and health-related stress were positively associated with fair/poor SRH. The adjusted OR indicated that older age (≥ 65 years) was significantly associated with increased odds of fair/poor SRH [OR = 1.17 (95% CI 1.09–1.27)], with the group of older adults with multimorbidity/high DS more likely to present fair/poor SRH [OR = 7.83 (95% CI 6.48–9.45)] than the group of older adults with only multimorbidity. Older adults living in rural areas were 36% more likely to present fair/poor SRH than older adults living in urban areas [OR = 1.36 (95% CI 1.24–1.50)]. While theoretically-plausible interactions were assessed, none were found (*p* > 0.05).

Table 4—Model 2 presents the results of the application of the Poisson regression model, which show that age, place of residence (rural/urban), years of education (≤ 9 years), and multimorbidity/high DS were positively associated with low life satisfaction. In contrast to those without multimorbidity, positive and significant association was found between older adults with multimorbidity/high DS and low life satisfaction [RR = 1.37 (95% CI 1.35–1.40)]. Only sex was negatively associated with low life satisfaction [RR = 0.96 (95% CI 0.95–0.97)].

## Discussion

Analysis conducted on a large amount of national-level data pertaining to older adults in Mexico revealed both a 25.8% prevalence of multimorbidity and that this prevalence increases with age. As the population ages, the prevalence of multimorbidity has increased in the majority of countries on a global level. Recent studies have reported a wide variety in the prevalence of multimorbidity, Wang et al., found that the prevalence of multimorbidity was 81.3% for older adults aged ≥ 60 years (Wang et al., 2020), one study from Taiwan in 2013 showed a prevalence of multimorbidity, which was 56.8%, 74.6%, and 82.6% in people aged 60–69, 70–79, and 80–89 years, respectively (Hu et al., 2019). In United Kingdom the prevalence of multimorbidity in participants aged 40 to 69 years was 19% (Zemedikun et al., 2018). The differences found in said prevalence may be due to the operational definition used, where multimorbidity has been operationally-defined from three main perspectives (Marengoni et al., 2011), while said differences may also be due to the age groups analyzed and the different methods of evaluating the presence of chronic conditions—be this via self-reporting or clinical diagnosis. Islas-Granillo et al., in a sample of older Mexican adults aged 60 and over, found a 27.3% prevalence of multimorbidity (Islas-Granillo et al., 2019), a result similar to that reported by the present study. The present study found a higher prevalence of multimorbidity in female than in male, which is consistent with the results of prior research (Im et al., 2022; Pache et al., 2015). A possible explanation for the higher percentage found is that women tend to be more conscious of their state of health and more informed about their medical conditions than men. Other explanation might be that females more aware of their own health status and had more healthcare-seeking behaviors, which might lead to a higher probability of being diagnosed with diseases (Thompson et al., 2016). However, gender is not the only explanation for the differences found, which can, in fact, also depend on social and economic determinants, which are, in turn, influenced by cultural conditions in society (Vlassoff, 2007).

The SRH is a subjective indicator most used for determining the general state of health and is also a good predictor of morbidity and mortality in older adults. The present study found that the combination of multimorbidity and high DS is related to a regular/poor SRH (OR = 7.83), while Camargo-Casas et al., found an association between multimorbidity and high DS and SRH (OR = 5.59) (Camargo-Casas et al., 2018). Sheridan et al., using data from the Survey of Health, Ageing and Retirement in Europe in 2013 and 2015 using data from the European Health, Aging and Retirement Survey in 2013 and 2015, they found that multimorbidity combinations that included high DS were associated with increased odds of reporting poor SRH (Sheridan et al., 2019). It is possible that combination of high DS to any chronic condition or combination of chronic conditions may be more disabling or associated with poor SRH than the combination of an individual condition (Sheridan et al., 2019). Multimorbidity and high DS have a negative impact on not only SRH but also other specific conditions, such as personal, behavioral, biological, and lifestyle factors (Whitmore et al., 2021).

It should be noted that, further to influencing the association between multimorbidity and SRH, DS may share predictive factors with multimorbidity and may, even, present a bidirectional relationship, namely being the consequence and not only the cause of negative SRH (Bustos-Vázquez et al., 2017). Ratner et al., found that the self-evaluation of the physical component has a greater influence than the mental component in SRH (Ratner et al., 1998), a gap that may show the complexity of the interrelations among these factors.

Stress is a normal physical, mental and emotional reaction, but it can be affecting especially for older adults. In the present study, it was observed that older adults with health-related stress are more likely to present a fair/poor SRH. This stress level among the older adults could negatively affect their health and well-being. Chronic diseases are the major causes of stress among the elderly (Khang & Kim, 2005). Likewise, long term stress can also lead to depressive symptoms among older adults (Jeon & Dunkle, 2009). Further, stress negatively impacts the body system’s ability to effectively respond to certain kinds of inflammation that lead to diverse health outcomes associated with aging (Lavretsky & Newhouse, 2012). Therefore, given the results obtained, it is of great importance to identify and obtain a deeper understanding of the existent relationship among these three variables (multimorbidity, DS, and SRH), as the combination of multiple chronic conditions can affect general and subjective health via not only DS but also multiple related variables (Ando et al., 2022). This is because the combination may increase the incidence of complications and adverse events, further, to having a negative impact on quality of life in the older adult population.

Life satisfaction is a concept that is frequently used to evaluate subjective wellbeing and reflect the feelings of the individual about their past, present, or future. The present study found that older adults with multimorbidity/high DS were 37% more likely to present low life satisfaction. Few studies that examine the relationship among multimorbidity, high DS, and life satisfaction are found in the literature, with these studies reporting contradictory results (Ando et al., 2022; Gan et al., 2022). The Korean Longitudinal Study of Ageing reveal that older adults with multimorbidity present higher DS levels and lower levels of life satisfaction (Lee et al., 2021), while Ando et al., found no association between multimorbidity and life satisfaction, but DS were related (Ando et al., 2022). Finally, Shang et al., in middle-aged and older people in China, they found a prevalence of DS was 30.3%, middle-aged and older people with chronic diseases experienced direct effects on DS (β = 1.011, *p* < 0.001). Chronic diseases had a more significant impact on the life satisfaction of middle-aged and older people (β = 0.037: *p* < 0.05) (Shang et al., 2023). There is a bidirectional relationship between DS and chronic conditions, due to the fact that behaviors adverse for health and the psychobiological changes associated with DS increase the prevalence of both chronic conditions and biological and physiological changes that impact the function of various systems of the body, leading to such symptoms as the following: increased inflammation; changes to heart rate and blood flow; stress hormone anomalies; and metabolic changes. These physiological symptoms can have a negative impact on the physical health of people with DS (Katon, 2011).

Although the behaviors associated with the lifestyles of older adults play an important role in their life satisfaction, it is often difficult for them to reach higher levels of life satisfaction as they age. The present study found associations between life satisfaction and age, sex (being a man), living in a rural area, and a low level of education. Similarly, research indicates that life satisfaction is influenced by various demographic and socioeconomic factors as well as ones related to health, physical and mental state, social support, social adjustment, and the number of multimorbidities (Dong et al., 2020; Wang et al., 2018; Banjare et al., 2015). Therefore, in order improve life satisfaction in older adults, it is of the utmost importance to not only pay attention to their general health but also analyze the social, cultural, and economic conditions in their environment with a view to having a positive impact on their quality of life.

## Limitations

First, the chronic conditions were evaluated by means of self-reporting, this has also been observed as a good indicator of health and mortality in the population, which the research could also have explored. Secondly, being a cross-sectional study, causality could not be evaluated and, moreover, some type of bias could also present. On the other hand, the strength is that it used a large representative sample of adults over the age of 50 years in Mexico (with both rural and urban representation) that enabled the evaluation of the impact of chronic conditions and various determinants of health on the process of aging in the population.

## Conclusions

The prevalence of multimorbidity was 25.8%. It was found that multimorbidity and high DS were associated with regular/poor SRH and low life satisfaction. Factors such as being ≥ 65 years old, living in a rural area, and a low level of education were associated with multimorbidity, high DS, and low life satisfaction in older Mexican adults.

The early identification of those factors related to multimorbidity, DS, and low life satisfaction is important in order to reduce possible health complications and improve people’s quality of life. Furthermore, the results obtained can be used to design and implement health policy that drives effective health prevention and promotion programs that both enable and guarantee wellbeing in old age.

## Data Availability

The data that support the findings of this study are openly available in Mexican Health & Aging Study at https://www.mhasweb.org/Home/index.aspx, reference number (grant number NIH R01AG018016).
